# Testing the quality of transformative science methods: the example of the Human Scale Development approach

**DOI:** 10.1007/s11625-021-00966-3

**Published:** 2021-05-20

**Authors:** Salina Spiering, María del Valle Barrera

**Affiliations:** 1 Helmholtz Centre for Environmental Research GmbH, UFZ, Leipzig, Permoserstraße 15, 04318 Leipzig, Germany; 2 Institute of Economics, Faculty of Economic and Administrative Sciences, Universidad Austral de Chile, Isla Teja, Valdivia, Chile

**Keywords:** Human scale development approach, Transformative science, Transformation research, Transdisciplinary methods, Quality criteria, Stakeholder involvement, Participatory processes

## Abstract

Scholars and funding bodies alike are increasingly calling for transformative research that delivers socially robust and impact-oriented outcomes. This paper argues that the Human Scale Development approach (HSDA) introduced by Max-Neef and colleagues in Latin America during the 1980s can serve as a method for transformative science (TSc). HSDA is both a theory and a participatory methodology and thus contributes toward fulfilling the objectives of TSc, which are scientific, practical and educational. In this paper, we begin by explicating how the fundamental human needs (FHN) approach of the HSDA can support scholars and practitioners alike in addressing complex sustainability challenges. We then refer to the methodological adaptations to the original proposal that we have previously developed to illustrate how Max-Neef's methodological approach might be further extended and to demonstrate how these changes can strengthen HSDA and make it even more useful for generating knowledge needed in sustainability transformations. To inform and support research that builds on the co-production of knowledge, we test HSDA with regard to TSc quality criteria and show how it adds value to the existing canon of TSc methods. To this end, we develop an analytical framework that can be deployed to assess the quality of TSc methods.

## Introduction: The Human Scale Development approach as a methodology for transformative science?

Tackling “wicked” environmental and societal problems that hinder sustainable development requires a reconsideration of how and what kind of knowledge is generated (Köhler et al. 2019; Bergmann et al. 2021). Caniglia et al. (2020) state that “sustainability science needs more systematic approaches for mobilizing knowledge in support of interventions that may bring about transformative change”. We argue that the Human Scale Development approach (henceforth HSDA) provides several entry points for addressing complex sustainability challenges and that it supports transformative change. First introduced in 1991 by Chilean economist Max-Neef et al. (1991), HSDA promises to offer a conceptual and methodological framework that enables researchers and participants jointly to “paint a picture” of current—or desired future—conditions based on the satisfaction of human needs. It provides a robust theoretical basis that is grounded on a broader understanding of “human needs” than neoclassical approaches such as the satisfaction of material needs, the marginal utility of consumption, the Unsatisfied Basic Needs Approach (Feres and Mancero 2001) or the World Bank’s income poverty measures (Altimir 1982). The intention pursued by Max-Neef and colleagues was to “make a theory of human needs understandable and operational for development”, as “human needs are discerned differently according to the ideological and disciplinary lens of the viewer” (Max-Neef et al. 1990:18). They devised the HSDA as a development approach whose purpose is not to increase productivity but to increase collective and individual well-being through the satisfaction of what they call Fundamental Human Needs, henceforth FHN (Guillen-Royo 2020). HSDA has been applied by various scholars in the context of environmental sustainability over the past ten years or so (Spiering and Barrera 2020). In our practical experience (ibid), HSDA provides a set of practical methodological guidelines for co-producing three types of knowledge needed to achieve sustainability transformations: (1) systems-knowledge (about what is), (2) target-knowledge (about what should be) and (3) transformation-knowledge (about how to get from where we are to where we should be) (Lys 1997; Kueffer et al. 2019). While the original methodological proposal of Max-Neef et al. (1990) mainly emphasizes the current and future states of a system (and thus provides systems- and target-knowledge), in previous work we have presented two adaptations of HSDA that focus additionally on providing transformation-knowledge (Spiering and Barrera 2020). In this paper, we present the HSDA theory of FHN along with our adapted methodological frameworks as valid methods for Transformative Science (TSc).

TSc was launched as a new paradigm nearly a decade ago to contribute to sustainability research with a normative orientation (Schneidewind and Singer-Brodowski 2013). It acknowledged that scientific outputs remain limited in addressing fundamental sustainability challenges. By contrast, TSc seeks not only to describe and analyse transformations but to further co-create and assess possible solutions and to carry out an educational mandate (Beecroft 2018; Bergmann et al. 2021). The World Social Science Report calls for transformative science “that facilitate[s] collaborative learning and problem solving, around concrete challenges and in specific social-ecological contexts” (ISSC/UNESCO 2013:9). Jaeger et al. (2011) emphasize that TSc is urgently needed to contribute to a democratisation of knowledge in an increasingly complex setting characterised by multiple compound problems.

Scholars generally agree that there are multiple methods for producing participatory knowledge, and many suggest that sustainability problems can best be solved by mixing and (re-)combining these methods (Wiek and Lang 2016; Wittmayer et al. 2018). To strengthen TSc research, the potential of these methods needs to be clarified and they need to be further developed and evaluated in relation to quality criteria (WGBU 2011; Belcher et al. 2016; Schneidewind and Rehm 2019).

To our knowledge HSDA has not yet been discussed as a method for TSc. While many methods for TSc exist (Wiek 2016), we are not aware of one that places reflection about human needs centre stage in the way HSDA does; we are convinced that such reflection substantially enhances the co-production of potential, evidence-based solutions and we therefore propose HSDA as a valuable method for inclusion in the TSc toolbox, complementary to existing methods.

The main question we explore in this article is the extent to which HSDA can serve as a method for TSc. This implies two further questions: is HSDA applicable at all in the context of TSc and, if so, what added value can it deliver to the existing canon of TSc methods? We seek to explore how HSDA can contribute to a mode of development based on reflection about the satisfaction of human needs and what implications this has for using it as TSc.

To address these issues, we examine (1) how HSDA is generally applicable in TSc and (2) how our adaptations meet TSc quality criteria. We do this by testing HSDA against TS quality criteria that we have compiled in an analytical framework to reflect on the research process and on scientific, societal and educational impacts. Our aim is to underpin the claim that HSDA fulfils the TSc quality criteria and can be used to address complex sustainability challenges. We outline the key elements of the approach and its implications for TSc, showing how HSDA delivers added value additional to other TSc methods. In doing so, we focus on the conceptual level and draw on our previous empirical research to illustrate, justify and exemplify our conceptual argument.

In “Background”, we introduce the HSDA perspective both conceptually and methodologically, providing descriptions of our two methodological adaptations. We then define TSc and present an analytical framework to assess the quality of TSc methods derived from the literature. In “Analysis”, we analyse the extent to which HSDA meets the quality criteria before outlining the added value of HSDA over and above other TSc methods. We close by presenting some limitations of HSDA for TSc and highlighting questions for further research.

## Background

In this section, we provide a conceptual and methodological introduction to the HSDA before introducing the concept and quality criteria of TSc.

### Introduction to HSDA

#### Conceptual background

In the 1980s, Max-Neef et al. (1986) devised the ‘Human Scale Development’ approach (HSDA) to provide an alternative model of development for Latin American communities to neo-classical models based on satisfying material needs (Pieterse 1998). While many human development theories have established indicator measurements (such as the human development index), HSDA provides both a taxonomy of human needs (theory) and a participatory process (methodology) through which communities are empowered to identify instances of deprivation as well as potential, according to how these needs are satisfied. HSDA is a people-centred development approach whose ultimate goal is to increase human well-being through the potential of individual stakeholders to meet their needs with appropriate strategies.

HSDA theory stands on three pillars: the satisfaction of FHN, the existence of organic interactions (e.g., between human beings), and the capacity for increasing levels of self-reliance. On this basis, both social groups and individuals are recognised as creators of their own future. HSDA fosters the active participation of people and thus promotes bottom-up decisions while empowering civil society. Max-Neef et al. (1991) developed a taxonomy of FHN based on their experiences in many small-scale workshops in Latin America and Europe. They identified nine axiological (values based) needs: subsistence, protection, affection, understanding, participation, idleness, creation, identity and freedom. These are realised through satisfiers, which exist in four existential categories: being (personal or collective attributes), having (institutions, norms), doing (personal or collective activities), and interacting (locations and milieus). Whereas FHN are considered to be universal, abstract and non-hierarchical, satisfiers differ from case to case and are dependent on culture, situation, education, institutions and other factors. Thus, every society, community or group adopts different styles and generates different types of satisfiers to meet the same FHN.

On this basis, Max-Neef et al. (1986) proposed a matrix of needs and satisfiers in which the columns contain needs according to the four existential categories, the rows list the FHN according to the nine axiological categories, and the resulting 36 cells contain the satisfiers. “The matrix of needs and satisfiers may serve, at a preliminary stage, as a participative exercise of self-diagnosis for groups located within a local space”. Through a process of regular dialogue—preferably with the presence of a facilitator acting as a catalysing element—the group may gradually begin to characterize itself by filling in the corresponding squares" (Max-Neef et al. 1991:37). While the FHN have a universal and normative character, the satisfiers are experienced in an individual and subjective way, though they are shared, constructed and changed socially, culturally and collectively. Such a matrix of needs and satisfiers can be used (and developed further) to take stock of existing goods and services and to highlight how they (or the social or natural systems providing these goods and services) contribute to or inhibit the fulfilment of needs. While some goods and services can be seen in some situations as strategies to meet one specific need (singular satisfier), they can simultaneously meet (synergic satisfier), fail to meet or even impede (inhibiting satisfier) several other needs at the same time. As a result, the satisfiers are the indicators of endogenous development, as they are felt and explicitly defined by each culture, time and place and are not imposed on actors as external elements. Focusing on needs and linking satisfiers to needs “allow[s] for the discovery of unexpected facets of a problem, thus increasing awareness about what [is] relevant” (Max-Neef et al. 1991:43). Although the original proposal provides a “matrix type’ as an example, the proposed methodology is based on the collective filling in of an empty matrix. The completed matrix is not normative but rather a heuristic device developed by a particular group or community at a given time. While the matrix is a methodological tool and not an end in itself, part of its potential is that it generates a reflective and critical attitude for diagnosis, planning and evaluation.

The matrix has since been used by academics and practitioners as both a theoretical framework in desk studies and a participatory tool with groups of people or communities (Spiering and Barrera 2020).

#### HSDA links needs with sustainability

Within the last decade, scholars have introduced the HSDA framework in the context of environmental sustainability and shown its potential to contributing toward sustainable development (Cruz et al. 2009; Jolibert et al. 2011; Guillen-Royo 2016, 2020; García Ochoa and Graizbord 2016; Lamb and Steinberger 2017; Vita et al. 2019; Kaltenborn et al. 2020).

The basic idea of the HSDA theory is that development is about people and not objects. Development and well-being are based on the “fulfilment” of FHN and not on “satisfaction” brought about by material consumption. Accordingly—and this is the key part of our argument—reflection on the nature of FHN is central to HSDA. To our knowledge, none of the other TSc methods highlights needs fulfilment in the way HSDA does. The current understanding of “needs” based on the Brundtland Report (WCED 1987) focuses almost exclusively on material aspects and thereby legitimates economic growth in a mono-dimensional way. As a response to this utility-based perspective, various scholars have developed approaches that accommodate a wider understanding of “human needs” (Sen 1984; Doyal and Gough 1984; Wiggins 1987). HSDA is one of these. Linking HSDA to sustainable development requires conceptual framings that enable those involved in normatively inspired processes to evaluate, design and implement sustainability transitions on the ground. HSDA focuses on human flourishing and provides a theory of human needs for development that goes beyond economic rationality, rather comprehending the human being as a whole (Cruz 2006). It distinguishes needs from satisfiers, meaning that needs are not seen in material terms alone but also in terms of basic psychological human needs. It thus functions as a tool that helps generate answers to the question: “what do I/we really need to live a good life?”. In this context Rauschmayer et al. (2012) and Guillen-Royo (2016) establish a link between needs and sustainability. HSDA enables a change in perspective on sustainable development by adding the categories of “being”, “doing” and “interacting” to merely “having”. It addresses not just material issues but brings in questions of what people are like, what they do and how they interact to fulfil their needs. A further contribution to sustainable development arises with the distinction between needs and satisfiers—needs are neither sustainable nor unsustainable, but satisfiers can be (for example, the need for freedom can be satisfied either by flying abroad or taking a walk in the countryside). HSDA provides the concept of synergic (bridging) satisfiers, which can be interpreted as “sustainable” strategies because they do no harm but support the fulfilment of several needs at once.

Jolibert et al. (2011) showed how the HSDA, as an anthropocentric human needs-based approach, can be widened to become a more global and ecosystemic one by reflecting the needs of non-humans (in this case otters) in an environmental fisheries conflict.

**Fig. 1:**
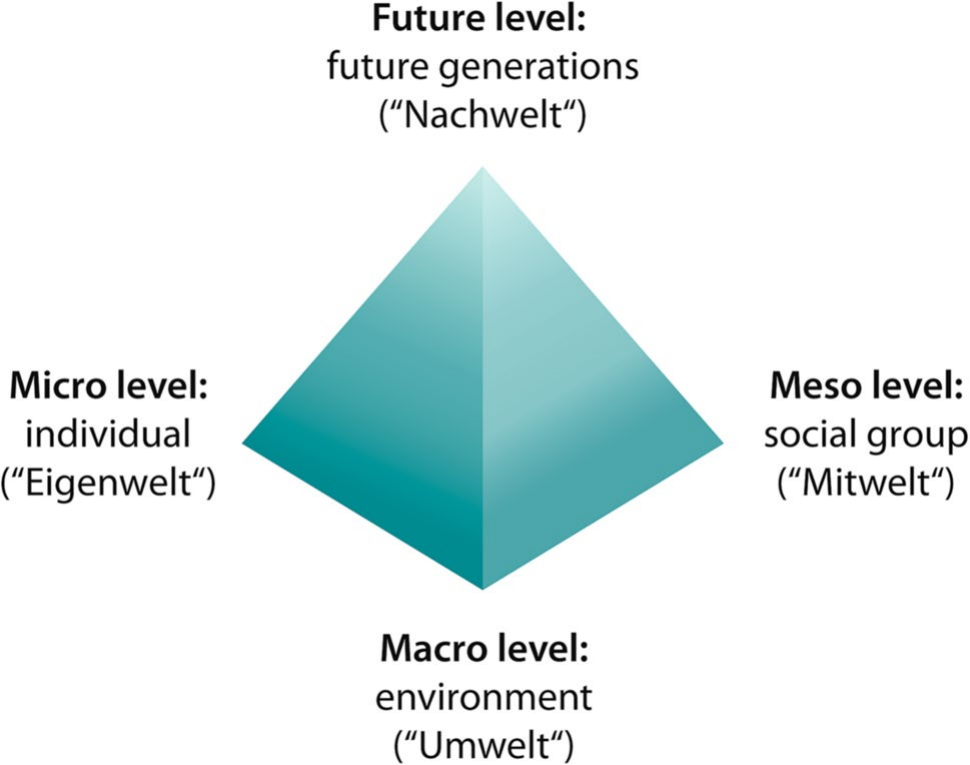
HSDA contributes to sustainable development by facilitating the synergic actualisation of needs on four levels

HSDA gathers people’s knowledge about the system in question and enables a synergic creation of shared knowledge about its actual and potential contributions to human (and non-human) well-being. “Needs are satisfied within three contexts: with regard to oneself (Eigenwelt); with regard to the social group (Mitwelt); and with regard to the environment (Umwelt)” Max-Neef et al. (1990:21). We add that by adopting a longer-term perspective on needs and sustainability, a fourth context can be added—posterity (Nachwelt) (see Fig. 1). To conclude our argument, then, HSDA informs TSc by helping those involved to move from the individual level of needs satisfaction to a collective dimension. By reflecting on the satisfaction of both human and non-human needs, it takes account of the individual (micro-level), the social group (meso level), the environment (macro level) and future generations (future level).

To our knowledge, HSDA has not yet been linked to the general scheme of TSc, either in general terms (e.g., contributing to the three knowledge types) or in more concrete terms (e.g., how it meets TSc quality criteria).

#### Adapted HSDA methodology

##### Applications of HSDA methodology

The original workshop methodology has been adapted and further developed in several ways and different fields, thus enhancing the original approach (Picón et al. 2006; Bucciarelli and Alessi 2013; Guillen-Royo 2020).

In a previous article, we presented two possible adaptations of the original methodological HSDA proposal and provided detailed guidelines for the facilitation of HSDA workshops (Spiering and Barrera 2020). Here we refer to this work to illustrate how these adaptations of the original HSDA proposal contribute toward generating TSc knowledge. The version developed by Barrera (Ibid 2020) is an adaptation of the original methodology generated in the learning environment of the HSDA Master’s programme at the University Austral of Chile (UACh) in close collaboration with Manfred Max-Neef. The version proposed by Spiering (2020) builds mainly on Guillen-Royo’s (2016) workshop proposal. Our particular contribution is to adapt the original HSDA and apply the new frameworks in various case studies. Between 2014 and 2019, the authors conducted a total of 18 workshops (10 conducted by Barrera in Chile and 8 by Spiering in Germany and Chile), each time following the procedures outlined below. For the purposes of this paper, we refer to two case studies, illustrated in “BOX 1” and “BOX 2” below. Rather than providing detailed case descriptions, we describe them only briefly as a means of underpinning our argument.

BOX 1: Case study of the Centre for Entrepreneurial Learning with vulnerable teenagers (Barrera 2017)

**Table:** 

The Centre for Entrepreneurial Learning (CEM) at UACh in Chile run a case study that was part of the “Youth Economic Participation Initiative” project (YEPI) (Tisch 2017). CEM supports innovative ways of educating and preparing young students for employment and entrepreneurship. Its programme is based on the belief that students can be powerful agents for change in their own communities (Hoyt et al. 2016). CEM seeks to encourage locally rooted leadership development and community-driven entrepreneurial projects as it “is not a typical university centre. […] Its commitment to Human Scale Development functions as a model for community development, while strengthening place-based culture and history” (Hoyt et al. 2016: 6). Within the YEPI project CEM convened four HSDA workshops between 2014 and 2016 with young people from different Chilean communities and backgrounds who came together to reflect on the potentials as well as the deprivations young people are facing in their communities, linking the local with the regional scale. In all, between 10 and 20 young people attended the workshops, which were facilitated by up to seven HSDA Master’s students with the support of one of the authors (Centro de Emprendizaje UACh 2015; Barrera 2017)

BOX 2: Case study with German Energy Cooperatives (Centgraf 2018)

**Table:** 

The second case study was part of the German EnGeno research project (Lautermann et al. 2017) in which members of German Renewable Energy Cooperatives (henceforth RECs) reflected on the challenges and potential of their own involvement, starting with their individual needs. The main research question was: how can the members of RECs be supported in their largely voluntary activities? The study’s rationale was therefore that a needs-based perspective might contribute towards developing new strategies to help the members of RECs individually to meet the challenges arising from their civic engagement. In three HSDA workshops, board members as well as active and passive members reflected on the challenges they experienced due to the rapid development of their REC as well as on the potential benefits of their engagement. The goal of the study was to support the REC members, most of whom are involved on an unpaid basis, in order to help initiatives to remain robust over the long term. Facilitating the development of new strategies helped the members of RECs individually to better meet the challenges arising from their civic engagement (Centgraf 2018)

##### Brief overview of HSDA adaptations in relation to the original methodological proposal

Here we offer a brief description of our two adaptations of the original proposal without comparing their differences and commonalities in further detail, as this is done in Spiering and Barrera (2020).

**Fig. 2:**
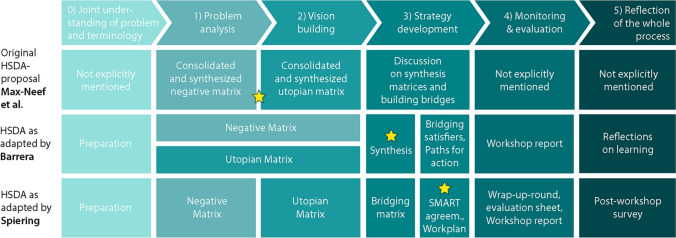
The original methodological proposal and two slightly differing HSDA adaptations

The methodological frameworks of our HSDA adaptations are structured according to Wittmayer and Hölscher’s (2016) proposal of six main phases within participatory research processes (Fig. 2). This structure was applied retrospectively to the original framework and the adaptations to facilitate an overview of differences and commonalities. Participatory research processes are divided into six main phases: (0) joint understanding of problem and terminology, (1) problem analysis, (2) vision building, (3) strategy development, (4) monitoring and evaluation and (5) reflection of the whole process. There are multiple examples of approaches that focus on phase one. “The latter parts (steps 2–5) often receive less attention and there are not enough methods to scientifically follow these steps and feed the outcomes back into the research process” (Wittmayer and Hölscher 2016:21). In both adaptations, we provide options for strengthening HSDA in this regard and demonstrate how the adaptations meet TSc quality criteria. Whereas the original proposal of Max-Neef et al. (1991) places emphasis on phases (1) and (2) we additionally focused on different ways of generating transformation-knowledge (phase 0) and phases (3)–(5)).

The original proposal by Max-Neef et al. (1991) does not include a preparation phase. Within our adaptations we included a phase 0, as we consider it important to collect information regarding participants’ expectations about the aims of the workshop and to formulate a shared definition of the problem.

Each of the three proposals uses its own concepts for filling in the matrix in a participatory manner to find all the factors that impede the actualisation of participants’ needs (phase 1) and the factors that support the group’s development in the best way possible (phase 2). Max-Neef et al. (1991) emphasize the negative and the utopian matrix (indicated in the figure by the yellow star). Barrera’s adaptation seeks to combine the problem analysis with the visioning phase in a single system analysis where negative and positive factors are collected at the same time. This process results in a negative and a positive matrix. Regarding strategy development (phase 3), in the original proposal, participants talk about bridging the negative and the utopian matrices to identify synergic satisfiers and how these can be implemented. However, this process is not presented in any further detail and nor is there any indication of what follows next. Barrera conducts a phase 3 where the previously identified satisfiers are then prioritised and validated or, on the contrary, revised or clarified; further the conversation is directed toward reflecting on the satisfiers as being endogenous, exogenous or synergic. This results in a synthesised matrix that forms the core of her approach. In Spiering’s adaptation, participants identify the key issues of the negative and utopian matrices to determine a number of synergic bridging satisfiers and build a new matrix using these bridging strategies. In contrast to Guillen-Royo’s (2010) model, the group undertakes a SMART analysis (**s**pecific, **m**easurable, **a**chievable, **r**elevant and **t**ime bound) and decides what kind of strategy can be implemented, by whom and by when. This results in a clearly defined timetable and work plan as one main outcome, in addition to an overview of all impeding factors, all utopian factors and the bridging satisfiers as strategies for further development.

Both Barrera and Spiering propose a phase 4 and phase 5, unlike the original version which mentions neither monitoring and evaluation nor a reflection of the process as a whole:

*Phase 4* In Barrera’s proposal, the workshop results are identified as potential pathways and guiding principles that participants possess and through which they aspire to satisfy their FHN. Finally, the data are interpreted by the researchers who are also part of the knowledge generation process. In Spiering’s adaptation, the workshops end with a face-to-face evaluation session and a written evaluation. As a follow-up, the participants are offered an extended workshop report that includes a compilation of all the results.

*Phase 5* Barrera reflects on the whole process in a project report. In the special case of the HSDA Master´s programme, these reports are presented by the researchers and students of the programme, as part of their learning and training as change agents. Spiering’s adaptation includes a post-workshop survey where information about the implementation of the work plan and comments concerning the methodology are gathered. For our own reflection on the whole process, we researchers evaluate the research diaries we have all kept from the beginning of the process.

With regard to our analysis of the appropriateness of HSDA as a method for TSc, these two adaptations are significant because they place an emphasis on phases (3)–(5) which are relevant to producing systems-, target- and transformation-knowledge. Our aim here is not to elevate our adaptations above other possible applications. Instead, we seek to present them as a supplement to TSc, highlighting those that we perceived as helpful and whose quality we were able to test using appropriate criteria.

### About TSc and how to assess its quality

#### Transformative science

In the following, we introduce TSc as an approach that exists beneath the wider umbrella of transformation research and is more encompassing than transformative research. In 2011, the German Advisory Council on Global Change (WGBU) launched transformation research as a reflexive and systemic approach whose main subject is “the global transformation towards a low-carbon society” within a democratic exploratory process (WGBU 2011: 332). Transformation research is described as an “emerging and common research perspective” (instead of a research field) that has widely been replicated and can “serve as catchment basin and integrator of diverse angles on societal change towards sustainability” (Wittmayer et al. 2018:6).

The WGBU differentiates between transformation research and transformative research. Transformation research is concerned with understanding and analysing transformation processes and is widely defined in terms of descriptive-analytical/knowledge first approaches that work with a “description and analysis of past, current, and future states” (Wiek et al. 2012:7). It thus focuses on generating conceptual knowledge. With the experimental turn in sustainability-related social sciences these “linear and technocratic solution approaches” (Bergmann et al. 2021) were recognised as being insufficient to address complex sustainability challenges (Overdest et al. 2010). Since then, scholars have increasingly pursued action- and practice-oriented research to produce “actionable knowledge” (Wiek et al. 2012; Caniglia et al. 2020). This is described as “evidence-supported guidance for practical application that has been tested in successful efforts to solving (or at least mitigating) a sustainability problem within the defined experimental setting” (Schäpke et al. 2016:47). It is sought to co-create robust context-specific solutions that are valid both inside and outside academia and actions necessary to address pressing social and environmental problems (Patterson 2015; Hölscher et al. 2021).

*Transformative research* is widely defined in terms of such process- and action-oriented approaches (Miller et al. 2014; Wittmayer and Schäpke 2014; Wiek and Lang 2016). Its goal is to “address problems of unsustainability challenges by inventing and assessing possible solutions and by creating related actionable knowledge, including strategies that can solve (or mitigate) certain problems” (Bergmann et al. 2021). Thus, transformative research complements descriptive-analytical approaches while also developing “evidence supported solution options” (Wiek and Lang 2016:32). However, being fairly new, transformative approaches are still far less represented within transformation research (Wiek et al. 2012; Feola 2015; Wittmayer et al. 2018).

Taking the differentiation between transformation and transformative research as their point of reference, Schneidewind and Singer-Brodowski (2013) proposed TSc as new paradigm that goes beyond transformative research and additionally encompasses transformative education and a focus on institutional change in the science system (Schneidewind and Singer-Brodowski 2016). TSc pursues a threefold aim: to generate new knowledge (scientific research objectives), to initiate and accompany transformation processes (practical objectives), and to stimulate and support learning processes (educational objectives) (Beecroft et al. 2018; Vogt and Weber 2020). These objectives form the basis of the analytical framework we present in “Analytical framework for assessing the quality of a TSc method” as a means to assess the quality of TSc methods. We put forward our argument within the TSc framework because we conduct transformative research in an environment of transformative education (with Master’s students and other sustainability science scholars). We argue that transformative research cannot be conducted without reflecting on institutional circumstances and the specific constraints of this kind of research within the scientific system; our hope is to induce changes by drawing attention to these.

TSc has been criticised for being too solution-oriented (solutionism) (Stock 2014; Strohschneider 2014; Grunwald 2018) and for potentially creating a de-politicised expertocracy (Meisch 2019). This debate has stimulated a process of clarification regarding the relation between science and society, the role of research in democracies, and funding conditions (Singer-Brodowski and Schneidewind 2019). Jaeger-Erben et al. (2018) warn that the intra-scientific debate on TSc is progressing so rapidly that the empirical knowledge base cannot keep pace. As TSc is in the developmental stage, they argue, it needs more rigorous systematisation and empirical TSc research practice that is methodologically and theoretically well grounded (ibid). With our analysis of HSDA as a method for TSc, we seek to contribute to sound methodological reflection on TSc methods, outlining its potentials and drawbacks for generating actionable knowledge.

#### Analytical framework for assessing the quality of a TSc method

Further empirical research designed on the basis of actionable knowledge is necessary because TSc lacks established, systematised approaches and sound empirical research practice (Wittmayer et al. 2018). On one hand, then, it may be that new, re-defined, or even re-discovered methods for TSc are needed; on the other, perhaps “the potential of existing methods [also] needs to be clarified” (ibid:18). Several methods have been developed for TSc and applied over the last few years (Wiek and Lang 2016; Wittmayer et al. 2018; Borner and Kraft 2018; Bergmann et al. 2021). In the German TSc debate, real-world laboratory research (Reallabore) has gained prominence (Wagner et al. 2016; Defila and DiGiulio 2019) as an “ideal-type […] of transformative research” (Schneidewind et al. 2016:10). What these methods have in common is that they build on participatory frameworks and are mainly of a qualitative character (Bergmann et al. 2021). Different facilitation techniques including creative, arts-based and visual methods, especially ones that promote dialogue, are increasingly being deployed within TSc (Defila and DiGiulio 2018; Caniglia 2020). They contain components such as participatory visioning, pathways and backcasting (Quist et al. 2011). Still few quality criteria have thus far been described and defined specifically for TSc, even though scholars insist it is crucial to develop TSc methods and quality criteria to (1) contribute to the recognition of co-produced knowledge among “traditional” scholars, (2) pave the way for a debate about how these might contribute to transparency and credibility in co-produced knowledge, and (3) support the research practice that would benefit from a TSc methods canon of proven quality (Wiek and Lang 2016; Stelzer et al. 2018; Defila and DiGiulio 2018). Existing disciplinary quality criteria and quality control indicators based on the present scientific paradigm (excellence, peer review and citation indexes, among others) are not sufficient for the evaluation of TSc, as they neglect the societal relevance and educational impact of TSc research (Wittmayer et al. 2018).

**Fig. 3:**
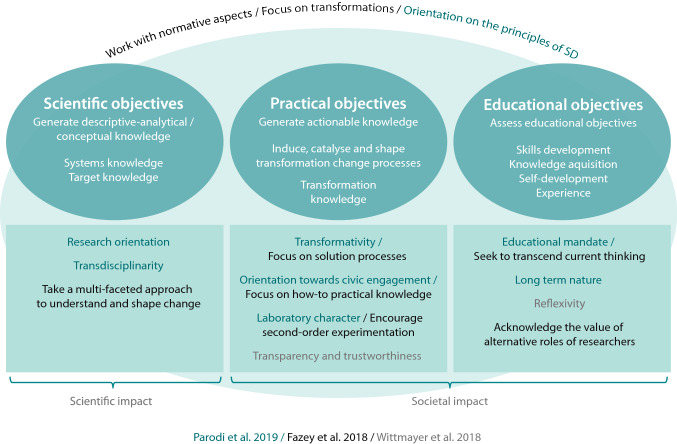
Analytical framework—assessing TSc methods that ideally display certain characteristics and thus contribute toward addressing three main objectives

While quality criteria in relation to transdisciplinary research methods have been discussed in the scientific literature for many years in general (Bergmann et al. 2005; Jahn and Keil 2015; Zscheischler et al. 2018) only few proposals are currently being debated with regard to a methodological quality assurance of TSc (Defila and DiGiulio 2018; Wiek 2016; Hölscher et al. 2021; Bergmann et al. 2021). As TSc is defined by characteristics like co-design, practical relevance, democratic knowledge production, normativity and catalysing role, quality criteria for TSc need to be based on these characteristics (Jaeger-Erben et al. 2018). For our exploration of the suitability of HSDA as a TSc method, we have developed an analytical framework in which we combine certain TSc characteristics (Parodi et al. 2018, 2019), essentials for second-order research (Fazey et al. 2018) and criteria (Wittmayer et al. 2018) to show how they contribute toward addressing the three objectives of TSc (Fig. 3).

##### Normativity

The overarching goal of TSc is to catalyse change for sustainability transformations, which requires an explicit normative positioning (Fazey et al. 2018). Several scholars highlight the need for suitable facilitation approaches that enable normative issues to be addressed when devising instruments for sustainable transformations (Fazey et al. 2018; Wamsler et al. 2020; Hölscher et al. 2021). Schäpke (2018) stresses the need to address the “normativity gap” within transition studies while Parodi et al. (2019) caution that it is important to stick to the characteristics of real-world labs, such as normativity and an orientation toward the principles of sustainable development, in order not to dilute the concept. Thus, it is vital that TSc methods contribute toward revealing underlying unsustainable practices and challenging unsustainable “structures, systems, mindsets and cultures” (Fazey et al. 2018:57).

Further, the central challenge of TSc lies in in its aim of combining scientific knowledge production about transformations with that of catalysing practical changes and facilitating learning processes (Beecroft 2018).

##### Scientific objectives

The scientific objectives of TSc are to create a scientific impact by generating, integrating, assessing and disseminating conceptual knowledge relevant to transformations (Wittmayer et al. 2018; Parodi et al. 2018, 2019). Considering the three types of knowledge mentioned above (cf. “Introduction: The Human Scale Development approach as a methodology for transformative science?”), TSc methods should generate systems-knowledge (to understand an issue, its dynamics and causal influences) and target-knowledge (about the desired future state of the system and why this state is desired) (Lys 1997; Parodi et al. 2018, 2019; Beecroft et al. 2018). In contrast to Beecroft et al. (2018), we assign transformation-knowledge to “practical objectives”, as this kind of knowledge not only describes options for change, but also contains potential for action (actionable knowledge). Gaining descriptive-analytical systems- and target-knowledge implies interdisciplinary as well as transdisciplinary approaches, as methods should contribute toward a multi-faceted approach to understanding and shaping change (Fazey et al. 2018).

##### Practical objectives

The practical objective of TSc is to achieve a social impact by co-producing actionable and transformation-knowledge (the third of the three types of knowledge) about the ways and means available of realising the desired state of a system in practice (Gaziulusoy and Boyle 2013; Wittmayer et al. 2018). Wittmayer et al. (2018) state that research should be judged according to its social impact. TSc methods should thus focus on solution-oriented processes suitable for “shaping the societal changes needed and for implementing solutions” (Fazey et al. 2018:57). Further, they should address critical questions about solutions and their implementation and thus generate “how to” practical knowledge that informs research. This requires practical engagement with civil society while acknowledging that practical knowledge is embodied and context specific (ibid). TSc methods should provide structured procedures and open spaces for experimentation, as creating change requires iterative learnings, thus the “laboratory character” of these processes should be acknowledged (ibid, Parodi et al. 2018, 2019).

##### Educational objectives

The third dimension of TSc seeks to achieve educational objectives. Beecroft (2018) suggests that educational objectives should be assessed explicitly in the planning, support and evaluation of projects and their respective methods. Researchers should consider, he argues, whether the participants (scientific scholars, students as well as practical and other stakeholders) in the TSc process are pursuing educational objectives for themselves or whether they are also seeking to educate others. Thus, methods should be evaluated in terms of the kind of educational objectives they help to achieve; above all, the educational mandate of TSc should be acknowledged (Parodi et al. 2018, 2019). Beecroft (2018) differentiates between skills development, knowledge acquisition, self-development and experience. To support these different kinds of learning, TSc processes should ideally be of a long-term nature (Parodi et al. 2018, 2019) and seek to transcend current thinking, as “many contemporary problems cannot be addressed by the same kinds of thinking that created them” (Fazey et al. 2018: 57). As part of this, reflexivity is seen as a crucial element of TSc that is frequently omitted (Finlay 2002; Pereira et al. 2020). Yet it is vital to reflect on the relation between researchers and practitioners, the researchers´ influence on the processes and outcomes as well as on their underlying (normative) assumptions (Wittmayer et al. 2018; Fazey et al. 2018). Even more important here is that researchers reflect on and value their different roles in TSc research processes (ibid, Wittmayer and Schäpke 2016). This includes introspection and a consideration of the researcher’s own background and normative orientation (Wittmayer et al. 2018).

**Fig. 4:**
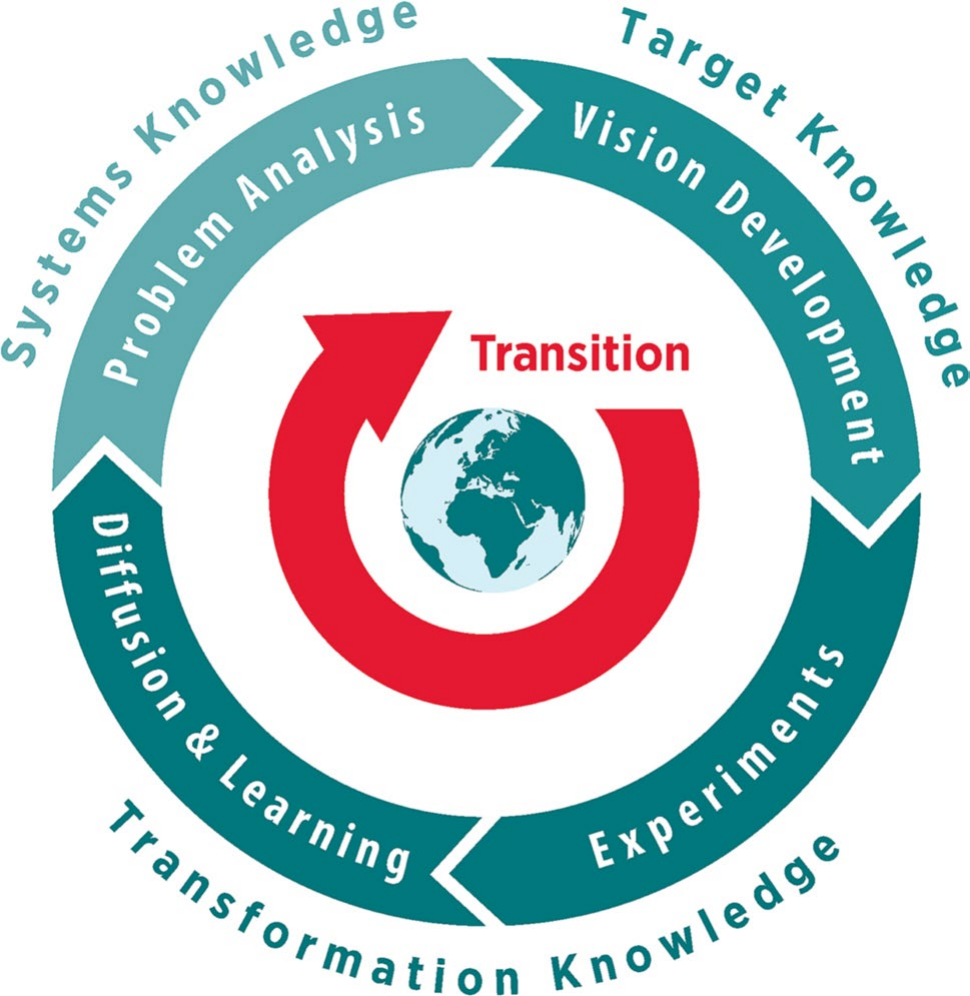
Transition-Cycle, source: German Wuppertal Institute (Bierwirth et al. 2017:31) (based on Loorbach 2010:173)

With the analytical framework presented here, we seek to show how HSDA embodies TSc characteristics and contributes to its three main objectives. To show how HSDA contributes toward co-producing the three types of knowledge for TSc, we will use the Transition Cycle (Fig. 4) presented by the German Wuppertal Institute based on Loorbach’s (2010) Transition Management Cycle as “a concept that enables transformation research to be linked to transformative research, that focuses on the principles of transdisciplinarity, and that aims to generate socially robust knowledge. As a concept ideal for practical research and a blueprint for research designs, the Transition-Cycle aims to take into account all three forms of knowledge” (Bierwirth et al. 2017:30). Given that the adapted HSDA is an appropriate tool, it is vital to ensure the scientific quality of the research to protect the credibility of TSc for sustainable development.

## Analysis

In this section, we analyse the extent to which HSDA meets the quality criteria for TSc methods presented above. To do so, we draw on early HSDA studies and also refer to our own published work (Barrera 2017; Centgraf 2018; Olivares-Aising and Barrera 2019; Spiering and Barrera 2020).

### Normativity: how HSDA re-orients sustainable development

Offering and conducting HSDA processes is about contributing to change in a certain community/group/system: it explicitly allows normative assumptions—as long as these are made transparent. HSDA was originally introduced to re-conceptualise the economic development process in terms of human well-being from a systemic perspective, and this entails a powerful normative component (Cruz et al. 2009). When the focus is on sustainability transformations, HSDA processes offer explicit normative orientations by conceptually linking needs with sustainability (cf. “HSDA links needs with sustainability”). Guillen-Royo (2020) promotes HSDA as a needs-based approach to consumption that is fundamentally normative “as consumption practices and behaviors are evaluated in terms of their contribution to human needs” (ibid: 118). Jolibert et al. (2014) address sustainable planning as a normative goal. They propose a definition of “sustainable satisfiers” to negotiate sustainable regulations and politics based on needs actualisation. In the RECs example, the normative objective was to support RECs as niche players within the German energy transition to strengthen their development (Centgraf 2018). The Chilean team operated under the normative framework of CEM (Hoyt 2016). Several scholars point out that it is essential for scientists to initiate deliberative learning processes with different societal actors to negotiate what sustainability means for specific situations (Schäpke 2018, Schneider et al. 2019). The HSDA concept of synergic needs actualisation provides a normative yardstick and allows to reflect on both existing sustainable development visions and on the creation of new ones that are context dependent. However, whether or not a process has been transformative can only be assessed ex-post. Thus, as method for TSc, HSDA can help to orientate research toward transformative change, but it should be cautious about claiming to generate normative results.

### Scientific impact

#### Research orientation

Scholars are increasingly applying and assessing the HSDA within research projects to gain conceptual knowledge about sustainability transformations and to publish their results in books or peer-reviewed journals (Gonzales 2010; Guillen-Royo 2016; Lamb and Steinberger 2017; Brand-Correa et al. 2018; Vita et al. 2019). In the RECs case, for instance, the results were fed into a scientific debate on energy cooperatives as niche players and change agents for sustainability transformations (Huybrechts and Mertens 2014; Brummer 2018; Centgraf 2018). With her HSDA research, Guillen-Royo contributes conceptual knowledge to the scientific discourse on sustainable consumption (Guillen-Royo 2020). These projects thus generate and reintegrate conceptual knowledge and reliable insights that can be taken up within science (Defila and DiGiulio 2019). In this way, they contribute “to the further development of scientific knowledge” (Altrichter and Feindt 2008:461).

#### Taking a multi-faceted and transdisciplinary approach to understanding and shaping change

Collaboration and co-production with diverse stakeholders in transdisciplinary settings is a core element of the HSDA, as groups are crucial to meeting individual needs and generating well-being, and vice versa. In the RECs case, the research team included people with a background in ecological economics, environmental psychology and geography, respectively, and they collaborated with REC board members, members and political stakeholders. Thus, multiple perspectives were included in the co-production of knowledge. The co-creation of synergic bridging satisfiers within HSDA processes includes multiple perspectives on development and furthers negotiation processes on how the fulfilment of needs shapes actions and decisions. HSDA functions as a pluralistic approach that recognizes diversity and offers options to negotiate multiple lenses and ways of understanding the world (Fazey et al. 2018).

#### Generating systems- and target-knowledge

HSDA provides useful entry points with recursive learning loops for the development of descriptive-analytical system- and target-knowledge (cf. Fig. 4). The original methodological proposal mainly identifies the deprivation present in a given society and thereby contributes to an understanding of the factors that impede development (systems-knowledge). This analysis gives rise to a sense of encouragement and constitutes a starting point for developing further strategies for transitions. The adapted workshop format devised by Barrera (2017) also serves analytical purposes and thereby helps generate systems-knowledge, but it goes further as well: by building a synthesising matrix and discussing bridging and synergic satisfiers, the process uncovers the potential that communities and groups have at their disposal (target-knowledge, phase 3, Fig. 2). HSDA processes as proposed by Spiering (2020) inform TSc research by providing system-knowledge using the negative matrix, in which all impeding factors are collected, and target knowledge through envisioning and collecting all those factors that enable optimal fulfilment of FHN. These system analyses and participatory visioning elements are complementary to other TSc methods like transition management (Wittmayer et al. 2018:16). Such an analysis supports an understanding of the system at hand and helps participants to envision a desirable future. Further transformation-knowledge can be build on this.

### Practical impact—societal impact

#### Transformativity/create actionable and transformation-knowledge/ focus on solution processes

HSDA processes open up transformative spaces in which relevant, context-sensitive and socially robust actionable or transformation-knowledge is engendered. Both HSDA adaptations above focus on solution-oriented processes and finish with action plans based on a reflexive dialogue about possible future orientations. In the RECs and the CEM case, a set of very concrete outcomes were generated along with applicable, context-specific knowledge and options. Barrera’s adaptation lays the groundwork for sustainable development strategies by discussing bridges between the synthesised matrices and reflecting on the satisfiers as endogenous, exogenous or synergic (transformation-knowledge). In Spiering’s adaptation, transformation-knowledge is generated through the elaboration of action plans for implementing strategies and mutual learning. Thus, conditions for change unfold, while the salience and accessibility of outcomes depends on the participants and on how they implement the strategies jointly developed during the process (Caniglia 2020).

#### Orientation towards civic engagement/focus on practical ‘how to’ knowledge

The HSDA adaptations focus on practical ‘how to’ knowledge and critically scrutinise potential solutions by reflecting on their nature as synergic or impeding satisfiers. In both cases presented above, the civil society actors perceived their ownership of the outcomes due to their strong involvement and the status of being experts in their own field. The socially robust outcomes of HSDA processes are firmly rooted in the self-reliance of the actors and thereby “change the way in which people are enabled to perceive their own potentials and capabilities” (Max-Neef 1990:52). On the individual and collective level, participants are empowered by reflecting on strategies that fulfil their needs and the needs of the group members and community. A remaining challenge—as for transdisciplinary impacts in general (Bergmann et al. 2021)—is to identify the social impact of HSDA processes more precisely. Here we can only make approximate statements about how change has been effected at individual, collective and system levels and how these change processes might be further catalysed (Pereira et al. 2020).

#### Laboratory character/encourage second-order experimentation

In its different applications, HSDA explicitly opens up spaces for experimentation: in the vision building and strategy development phases, possible development options are co-produced. In a participatory action research project, Guillen-Royo (2014) accompanied workshop participants in a Peruvian case study to organise and implement an organic vegetable garden and a parents’ school as they had formerly ranked these strategies as most synergic bridging satisfiers for needs actualisation. This learning by doing process illustrates how an HSDA process may support learnings for larger and more complex activities and may produce evidence about and action for solutions (Fazey et al. 2018). For this purpose, HSDA provides a well-structured, iterative learning process to enhance self-empowerment and create change. It does not suggest a particular development trajectory but focuses on process-based change towards a community that would be desirable for its citizens. Defila and DiGiulio (2018: 9) suggest the following preconditions for breaking new ground and experimenting: the actors involved must be willing and able to leave their own comfort zone, to detach themselves from their thinking and decision-making mechanisms and to think and trying out things they have not thought and tried before. The research setting, in turn must provide the actors with a framework that enables them to do things that they would otherwise not dare to try. This requires the creation of spaces for experimentation that allow all actors involved to try the unfamiliar. In the RECs as well as in the CEM cases, both of these preconditions were given: participants took the plunge to reflect their personal and collective needs and HSDA scholars provided what Pereira et al. (2020) call “safe-enough-spaces” within transformative learning environments. These spaces acknowledge the vulnerability of participants who might be putting themselves at risk by participating in the first place or who may be challenged by having to “unlearn and relearn” their own assumptions and points of view.

#### Transparency and trustworthiness

Our adaptations provide structured and replicable sequences of steps that contribute to the transparency of the processes and ensure the credibility of the research (Wiek and Lang 2016). Clarification of expectations and goals in advance is crucial to communicate clearly the purposes for which HSDA is applied. In general terms, van der Hel (2018) recommends humility regarding the capacity of science to provide solutions. Jaeger-Erben et al. (2018) argue that the transformative implications of an intervention may only be assessed retrospectively; they appeal for more restraint concerning the outcomes of transformative processes. In the cases presented here, the time frames were set very clearly, as were the goals, namely, to provide expertise on the methodology rather than solutions for future development. In the RECs case, this prompted some frustration, as some participants had expected they would receive advice from experts (the researchers) on how to further develop their REC. Thus, communication of the potential and limitations of the process is essential. Finally, transparency was established by providing access to the methodological procedures and results through workshop reports, including reports in which the process was reflected upon. To foster the trust and confidence needed to engage in open and honest dialogue, a sense of shared identity with the participants is established that validates all the participants’ right to speak for and on behalf of others, as was the case in the CEM study.

### Educational impact

#### Educational mandate/seek to transcend current thinking

HSDA processes as we propose them constitute a self-reflexive tool that, beginning with problem definition and a joint understanding of terminology, promotes a dialogic process containing numerous elements of mutual learning. It thus achieves some significant educational objectives (Spiering and Barrera 2020). In the case of CEM, educational objectives were explicitly planned, supported and evaluated before, during and after the research process. Participants as well as scholars and students developed new skills throughout the HSDA processes (e.g., participants got to know a new method of reflection, students improved their facilitation skills). All the participants acquired new knowledge: systems-, target- and transformation-knowledge. Self-development processes were triggered by the process of reflecting on individual needs. According to Horlings et al. (2020), research aimed at supporting transformation can change the researchers themselves. They point to action research, “where these types of changes are called ‘process-impacts’ which include changes in modes of collaboration, relationships, everyday practices and worldviews” (ibid: 471). The HSDA process provides spaces for open dialogue in which all actors experience the co-production of actionable knowledge. This is something Wamsler et al. (2020) deem essential to mindset changes to transcend current thinking. These spaces for reflection enable the contestation of prevailing views, needs and interests through individual and social learning processes. In this way, tacit knowledge that is “developed by doing, and embedded in skills, expertise and values” was made explicit (Caniglia 2020:50).

#### Long-term nature

The cases presented above comprised just one or two workshops and the respective pre- and post-workshop surveys. Follow-up work went further, addressing community engagement. Out of her experience with long-term participatory action research processes Guillen-Royo (2016) warns that one-off workshops may lead to disempowerment within collaborative endeavours. Following Pereira et al. (2020) transformative spaces need continued processes of engagement. In this regard, then, the case studies were not ideal regarding their potential impact, but depending on the financial and temporal resources available, HSDA processes can be extended over prolonged periods of collaboration, thus enhancing their impact. In a similar vein, Parodi et al. (2018, 2019) as well as Defila and DiGiulio (2020) emphasize that real-world laboratories in TSc are demanding and need time to initiate, establish, and evaluate, reflecting profound self-reflective learning processes. This requires not only different funding schemes for TSc research projects, but also a different kind of policy support (Muhonen et al. 2020).

#### Reflexivity

As a process for reflecting on needs, HSDA provides several entry points for establishing a reflexive practice. As the original HSDA proposal places no emphasis on scientific reflexivity, the research teams involved in developing the later versions deliberately established spaces for it. We reflected on how we intervened in the process, whether it be with the participants, within the team (in the CEM case with students of higher education) or when writing research diaries.

Like other TSc methods HSDA triggers counter-hegemonic processes and interpretations of results, as also happens with the people (workshop participants) who analyse, discuss, interpret and construct the satisfiers (Hölscher et al. 2021). The degree of intervention of the researcher is reduced to that of facilitator of the process, thereby undermining the asymmetric relationship between researchers and researched and potentially inverting the logic of power of scientific knowledge. This is in line with Ejderyan et al. (2019) assertion that critical Social Science and Humanities researchers must provide methods that sharpen the critical skills of all those involved. There are limits to this, however, as the complexity of the approach requires trained facilitators: the participants are thus dependent on a certain scientific expertise.

## Acknowledge the value of alternative roles of researchers

HSDA processes provided by research teams demand that researchers adopt flexible roles; this role switching brings many challenges and is regarded critically as it places considerable demands on the researchers (Jaeger-Erben et al. 2018; Scholz 2017; Defila and DiGiulio 2019). Simultaneously, as self-reflexive researchers, critically reflecting on and evaluating roles is vital when shaping research, actions and outcomes (ibid). In the RECs case and in the CEM case, the researchers affected the process on multiple levels, not only as facilitators but also by initiating the workshops, being part of the selection of workshop participants, introducing HSDA theory as a background to the reflection process, facilitating discussions and helping to compile and present the outcomes. Additionally, the authors were trained beforehand as professional facilitators and were thus able to prevent a sense of being overloaded with tasks—something Jaeger-Erben et al. (2018) point out as a limitation for researchers who are not facilitation professionals. Nonetheless, challenges still arose in terms of distinguishing between the different roles and in terms of expectations concerning these roles. Fazey et al. (2018) argue in favour of acknowledging the value of researchers’ different roles and making the challenges associated with this explicit. In an HSDA case study in Australia, Cuthill (2003:475) engaged an external facilitator to “minimise any influence or direction-setting by the researcher”. However, Max-Neef et al. (1991:100) stress the value of the facilitator’s personal involvement, and we agree when they state “no understanding is possible if we detach ourselves from the object of our intended understanding. Detachment can only generate knowledge, not understanding.” Although there is this overall claim of reflexivity in transformation research, up to now there has been scant dissemination of self-reflexive processes when educating students. In the CEM case, CEM as a university actor provided spaces for students to undertake a self-reflective process regarding their own values and motivations. Even here, though, this process of reflection was not embedded within a quality-assured, proven systematic procedure. We therefore agree that, ideally, such self-reflexive processes should be an integral part of academic education, to enhance researchers’ awareness of the issues involved and their capacity for dealing with them (Schneider et al. 2019; Hölscher et al. 2021).

By scrutinising the HSDA in terms of the TSc quality criteria outlined above, we have shown that it serves as a method for TSc that contains several TSc characteristics and contributes to the three objectives of TSc.

## Concluding discussion—added value of HSDA for TSc

In this final section, we discuss the advantages presented by HSDA in addition to other TSc methods, and what its drawbacks are.

### Added value of HSDA for TSc

We have shown above that the HSDA contains several TSc characteristics and has the potential to add to the TSc methods canon. In doing so, we have contributed toward a conceptual and empirical-based understanding of how HSDA can inform TSc. Jaeger-Erben et al. (2018) conclude that the innovative potential of TSc lies in the principles of co-design and co-production, democratic knowledge production, its holistic nature, and reflexivity; we argue here that this holds true for HSDA as well. Additionally, we derive three major advantages of HSDA from our analysis that we have not found in other methods in this combination: (1) HSDA contributes toward achieving the three main objectives of TSc while generating all three types of knowledge; (2) HSDA provides an explicit normative compass for TSc by linking needs with sustainability; and (3) by including reflection on needs and focusing on human flourishing, it contributes to a shift in mindset and reveals where the current scientific system needs to be transformed to support research for sustainability.

#### HSDA is highly flexible to acquire scientific, practical and educational goals

**Fig. 5:**
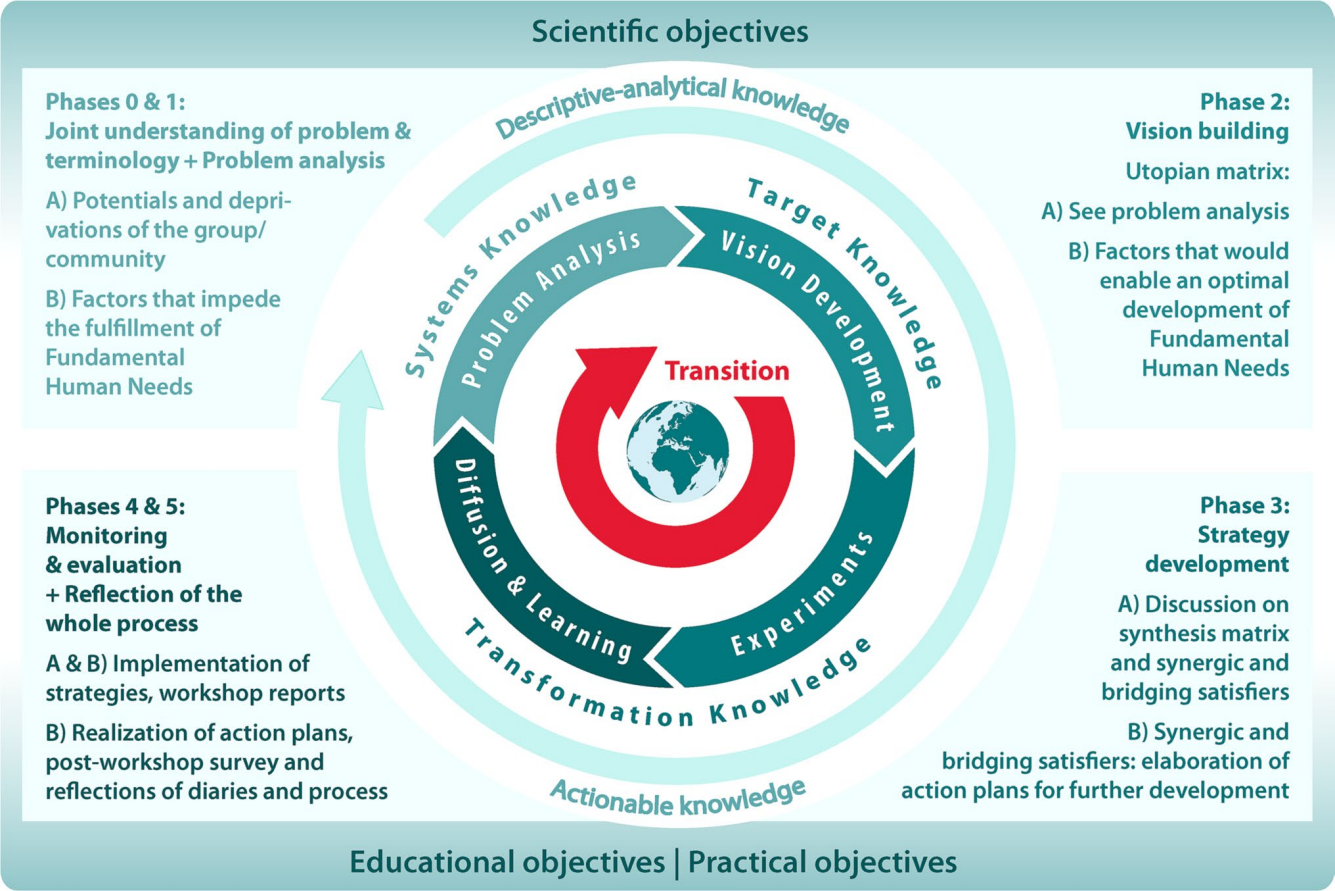
HSDA processes as proposed by Barrera (**A**) and Spiering (**B**) contribute toward generating knowledge for TSc and achieving TSc objectives ( adapted from Transition-Cycle, source: Wuppertal Institute (Bierwirth et al. 2017:31))

One particularity of the HSDA, when adapted as outlined above, is its potential to generate systems-, target- and transformation-knowledge equally and thereby (potentially) achieve all three TSc objectives. It thus addresses multiple dimensions, and this large degree of flexibility enables researchers and practitioners alike to focus on each of the six phases on the six-phase framework according to the aim of the process (Fig. 2). HSDA processes generate descriptive-analytical knowledge that detects unsustainable practices, mindsets, institutions and personal or collective activities of a given group and thus nurtures critical thinking and may have a scientific impact (Fig. 5).

HSDA makes actionable knowledge explicit through collaborative experimentation and mutual learning; it nurtures cultures of responsibility and may have a practical impact. Finally, HSDA processes as learning environments nurture empowerment and agency and (potentially) generate educational impacts (Kueffer et al. 2019). We conclude that defining problems jointly results in robust application-oriented outcomes (negative matrix); jointly developing visions results in empowerment (utopian matrix), and co-creating development strategies results in long lasting, sustainable solutions (synergic-bridging satisfiers). Thus, the original HSDA proposal and both adaptations in particular provide a highly suitable tool for encouraging creativity, empowerment and solidarity while generating descriptive-analytical as well as actionable knowledge and learning essential for TSc. The flexibility of HSDA ensures that knowledge production, or rather understandings, arise in what Bierwirth et al. (2017:21) call “recursive learning processes”. This is in line with Jaeger-Erben et al. (2018) who suggest a Grounded Theory of transformation-oriented research. Thus, recalling the criticism of “solutionism” (Strohschneider 2014), we follow Scholz’s proposal and classify HSDA outcomes as socially robust options that acknowledge “the specific constraints (including democratic compromise) under which real-world decisions are made” (Scholz 2017:12).

#### HSDA provides an explicit normative compass for TSc

With its unique link between needs and sustainability, HSDA offers a special normative compass for TSc. HSDA provides a valuable entry point for normative TSc research as it interprets synergic bridging satisfiers as sustainable strategies (Guillen-Royo 2016). Conceptually linking HSDA (that is: reflections on FHN) with sustainability opens up new avenues for focusing on sustainable development and putting it into practice (Rauschmayer et al. 2011; Rauschmayer and Omann 2015). Reframing sustainable development in line with a broader understanding of needs affects personal and policy-related decisions alike (Guillen-Royo 2016). Synergic bridging satisfiers can be considered as “sustainable” not only in relation to environmental issues, but also to social sustainability issues (Pelenc 2014; Olivares-Aising and Barrera 2019) and economic sustainability challenges (Guillen-Royo 2016; Göpel 2016). So far, this is an assumption, and we cannot claim that all synergic strategies are inevitably sustainable. The theoretical foundation of HSDA may be better integrated within sustainable development debates. It would certainly be worthwhile building further on the work of Pelenc (2014), who combines HSDA with Sen’s capability approach to strengthen HSDA’s theoretical foundation. Further reflection is needed regarding the outcomes of normatively oriented HSDA processes.

#### Reflection on FHN within HSDA induces mindset shifts that are also necessary for changing the scientific system

Recent literature on mindset shift stresses the need for a shift in paradigms and individual worldviews to face sustainability challenges (Göpel 2016; Wamsler et al. 2020). In this regard, Omann and Rauschmayer (2011:145) state that “a transition towards SD requires that actors start by looking into their inner side, reflecting upon one´s values, feelings, world views and being open and prepared to develop”. HSDA enables such reflection on multiple levels: it provides not only knowledge necessary for transformations, but also scientific understandings of a process based on reflection about FHN as well as individual and social learning processes. This inclusion of human feelings, values and needs provides new perspectives for TSc, as reflection occurs among participants and scientists alike. Pereira et al. (2020) call these processes of joint knowledge production that include consideration of emotions and empathy “humanizing solutions” that are socially relevant, personal and political**.** In a similar vein, HSDA processes may contribute toward transforming the science system itself, which is one objective of TSc**:** in the CEM case, students were educated and trained in HSDA theory and practice, while the other author has advised and trained other researchers to disseminate knowledge about practical HSDA applications (de Schutter et al. 2019). The science system will not be challenged by single projects, but nonetheless the very implementation of TSc projects has an impact on the science system itself (Moser 2016; Vogt and Weber 2020).

### Limitations and future research

In addition to some of the above-mentioned drawbacks to HSDA, we now outline some further limitations associated with conducting HSDA processes as well as more general challenges and implications when applying it as TSc.

With regard to the process of reflection on needs, it was clear that discussion at the personal level sometimes prompted silence, discomfort or even unease among participants. In this respect, it is all the more important to “create a safe learning space for learning and trust building”, but also to “embrace discomfort as a learning opportunity” (Hölscher et al. 2021).

Another highly topical challenge for conducting participatory research in general is the current coronavirus pandemic, which is making it difficult to create open spaces for dialogue (Engler et al. 2021). Within this context, the authors have started to experiment by conducting HSDA processes with students of higher education in virtual settings. Future efforts should be undertaken to test the empirical efficacy and robustness of virtual and/ or hybrid formats.

HSDA processes are limited regarding the continuity of the iterative learning process; although “risky” and “time-consuming”, the value of prolonged collaborations should be acknowledged (Jaeger-Erben et al. 2018:64). Several authors have begun demanding appropriate funding policies and respective project design for longer term TSc and transformation research (Caniglia et al. 2020; Defila and DiGiulio 2020; Bergmann et al. 2021). The highly aspirational threefold objective of TSc places considerable demands on all those involved (Bergmann et al. 2021; Defila and DiGiulio 2020): “this requires special and careful attention with regard to dealing with issues of legitimacy, a good management of expectations, and a reflective balancing of opportunities and threats” (ibid:64). Special attention should be drawn toward ensuring that HSDA processes can occur with no prejudgement of the outcomes, in order not to fall into the trap of believing that HSDA outcomes must necessarily be “transformative” (Jaeger-Erben et al. 2018).

Thus, further emphasis needs to be placed on reflecting about the outcomes of normatively oriented HSDA processes, developing further systematisations for HSDA reflections, and grounding HSDA theoretically in TSc. Future research could fruitfully reflect on related approaches by means of the blueprint presented here. For all the flexibility of the approach, however, reflection on the roles of the researchers and their impacts, values and objectives requires deeper analysis; in this article, we have referred only briefly to the issue of reflexivity and inner change (Parodi and Tamm 2018). More documentation of and experimentation with HSDA applications is needed to further develop the approach as a tool for TSc. To boost the uptake of our versions of HSDA, it would be feasible to publish open-access facilitation guidelines on platforms such as the USYS TDLab toolbox. The presented analytical framework can be applied to assess the quality of other TSc methods accordingly.

We conclude that our adapted HSDA methodologies inform TSc by being grounded in a theoretical basis, being highly flexible and normatively oriented, and by linking needs with sustainability. With its unique perspective on fundamental human needs and respective synergic satisfiers, HSDA provides alternative ways of understanding and catalysing sustainability transitions and inducing mindset shifts. We encourage other researchers to apply the adaptations presented here and to experiment with their valuable main components. HSDA is not presented here as the sole problem solver, but it may constitute one approach for catalysing actionable knowledge for sustainability transitions. We invite critical reflection on these issues and hope for further empirical testing and development in support of TSc on the basis of shared reflection on a human scale.
